# Nondestructive protein sampling with electroporation facilitates profiling of spatial differential protein expression in breast tumors in vivo

**DOI:** 10.1038/s41598-022-19984-x

**Published:** 2022-09-23

**Authors:** Edward Vitkin, Amrita Singh, Julia Wise, Shay Ben-Elazar, Zohar Yakhini, Alexander Golberg

**Affiliations:** 1 School of Computer Science, Reichman University (IDC Herzliya), Herzliya, Israel; 2 Porter School of Environment and Earth Sciences, Tel Aviv University, Tel Aviv, Israel; 3 Computer Science Faculty, Technion, Haifa, Israel

**Keywords:** Cancer imaging, Cancer prevention, Cancer, Engineering, Biomedical engineering

## Abstract

Excision tissue biopsy, while central to cancer treatment and precision medicine, presents risks to the patient and does not provide a sufficiently broad and faithful representation of the heterogeneity of solid tumors. Here we introduce e-biopsy—a novel concept for molecular profiling of solid tumors using molecular sampling with electroporation. As e-biopsy provides access to the molecular composition of a solid tumor by permeabilization of the cell membrane, it facilitates tumor diagnostics without tissue resection. Furthermore, thanks to its non tissue destructive characteristics, e-biopsy enables probing the solid tumor multiple times in several distinct locations in the same procedure, thereby enabling the spatial profiling of tumor molecular heterogeneity.We demonstrate e-biopsy in vivo, using the 4T1 breast cancer model in mice to assess its performance, as well as the inferred spatial differential protein expression. In particular, we show that proteomic profiles obtained via e-biopsy in vivo distinguish the tumors from healthy breast tissue and reflect spatial tumor differential protein expression. E-biopsy provides a completely new molecular sampling modality for solid tumors molecular cartography, providing information that potentially enables more rapid and sensitive detection at lesser risk, as well as more precise personalized medicine.

## Introduction

Based on molecular profiles of tumors and other tissues, personalized medicine aims to optimize medical care and preventative measures on an individual patient basis. In cancer therapy and care, a clear potential advantage has been demonstrated to the personalized approach as compared to traditional therapies^1–3^. Accurate diagnosis is a critical component of personalized medicine. An important component of molecular diagnostics in patient samples, including tumors, is the profiling of DNA, RNA, proteins, glycans, or metabolites, to identify molecular biomarkers that are predictive of tumor type^4–7^and of potential patient response^8,9^. To enable profiling of a solid tumor with a known and accessible location in the body, current methods use tissue biopsy, which involves the physical resection of a small tissue sample. This procedure leads to localized tissue injury, bleeding, inflammation, neural damage, and stress^10,11^, the effects of which are not well understood^12^. Moreover, standard tissue biopsy could increase the potential for tumor growth and metastasis^12–14^. In addition, because of the negative effects, only a few biopsies can be performed at a time, limiting the scope of the spatial mapping of the sampled site, and leading to misdiagnosis if the tumor is missed. Furthermore, some authors even concluded that due to solid tumor heterogeneity, information from a single biopsy is not sufficient for guiding treatment decisions^15,16^.

Indeed, recent literature identified the absence of efficient technologies for characterizing tumor molecular heterogeneity^17^ as a major limitation of the personalized medicine approach in cancer^18^. Significant genomic evolution often occurs during cancer progression, creating variability within primary tumors as well as between the primary tumors and their metastases^16,19–21^. Recent studies have shown that while a positive result (both successful biopsy and a decisive detection of markers) appears to reliably indicate the presence of the high-risk disease^15^, a negative result does not reliably rule out the presence of high-risk clones^22^. This is partly because a harvested tissue sample may not capture the most aggressive clone of a given tumor or tumor site^15,23–25^. Despite the significant improvement in molecular characterization technologies in recent decades, thanks to the introduction of new high-resolution sequencing and bioinformatics methods, these technologies remain limited by tissue sampling methods^16,26^. Thus, tissue sampling constitutes a critical limitation of personalized medicine^15,16,27^. New approaches to probing and profiling several regions in the tumor at the molecular level, termed molecular cartography, are expected to be useful in this context^28,29^.

Emerging technologies that could enable spatial molecular cartography in vivo include mass spectrometry coupled to electrosurgery (iKnife)^30^, laser ablation^31^ and liquid/liquid extraction^32^ (MasSpec Pen) are currently under advanced investigation. Yet, these technologies are still tissue destructive, as molecular sample extraction is achieved by tissue ablation during direct ionization.

To address these issues, and to extend the state-of-the-art of technologies that will potentially enable precision diagnosis and therapy, we developed a novel approach to molecular tissue sampling using electroporation^33^. Electroporation-based technologies have been successfully used to non-thermally irreversibly or reversibly change permeabilization of the cell membrane in vivo, enabling a wide set of applications ranging from non-thermal tumor ablation to targeted delivery of molecules^33^. We and others previously developed protocols for targeted delivery of electric field to tissues to induce focused electroporation at predetermined regions in organs^34–39^. We also showed, that different from other tissue ablation modalities, electroporation ablation preserves extracellular matrix and can lead to scarless regeneration^40,41^. In the previous work we studied the kinetics of molecular diffusion out of cells following electroporation with a needle electrode^42^. More recently, we showed that electroporation technologies selectively extract proteins and ash from biomass^43–45^. Although electroporation has been used to deliver molecules to tissues and to ablate multiple tumors and metastatic sites, to the best of our knowledge it has not so far been proposed for extracting molecules for tissue molecular profiling, including tumors in vivo.

The goal of this work is to test molecular harvesting by electroporation (e-biopsy) in vivo and to assess the spatial differential expression at the proteomic level, observable through this novel sampling method. We also compare the proteomic molecular profiles obtained through e-biopsy with state-of-the-art solid tissue lysis buffer extraction. In particular, we show that proteomic profiles obtained by e-biopsy from 4T1 mice tumors in vivo are tissue specific, consistent, reflect tumor protein expression heterogeneity, and align with proteomic profiles obtained using standard lysis buffers from excised tissue samples.

Our approach to solid tissue characterization, as described herein, differs substantially from needle or other excision biopsy approaches, which require tissue resection, as well as from liquid biopsy. The latter only measures an average biomarkers profile of the entire organism, and cannot provide precise spatial sub-clonal information. It is also, obviously, limited to the molecular content accessible in the patient’s circulation system. E-biopsy, when used in combination with in situ electrodes^46–48^, potentially expands the opportunity for capturing spatial clonal variations. Moreover, due to electroporation’s minimally tissue destructive nature^41,49,50^, e-biopsy potentially facilitates multiple sampling/probing, and thereby higher-resolution spatial molecular cartography of tissues at the macroscale. E-biopsy can thus enable a new type of diagnostic approach for spatial molecular tumor mapping in vivo that is not currently possible.

## Results

### E-biopsy for molecular harvesting in vivo

**Figure 1:**
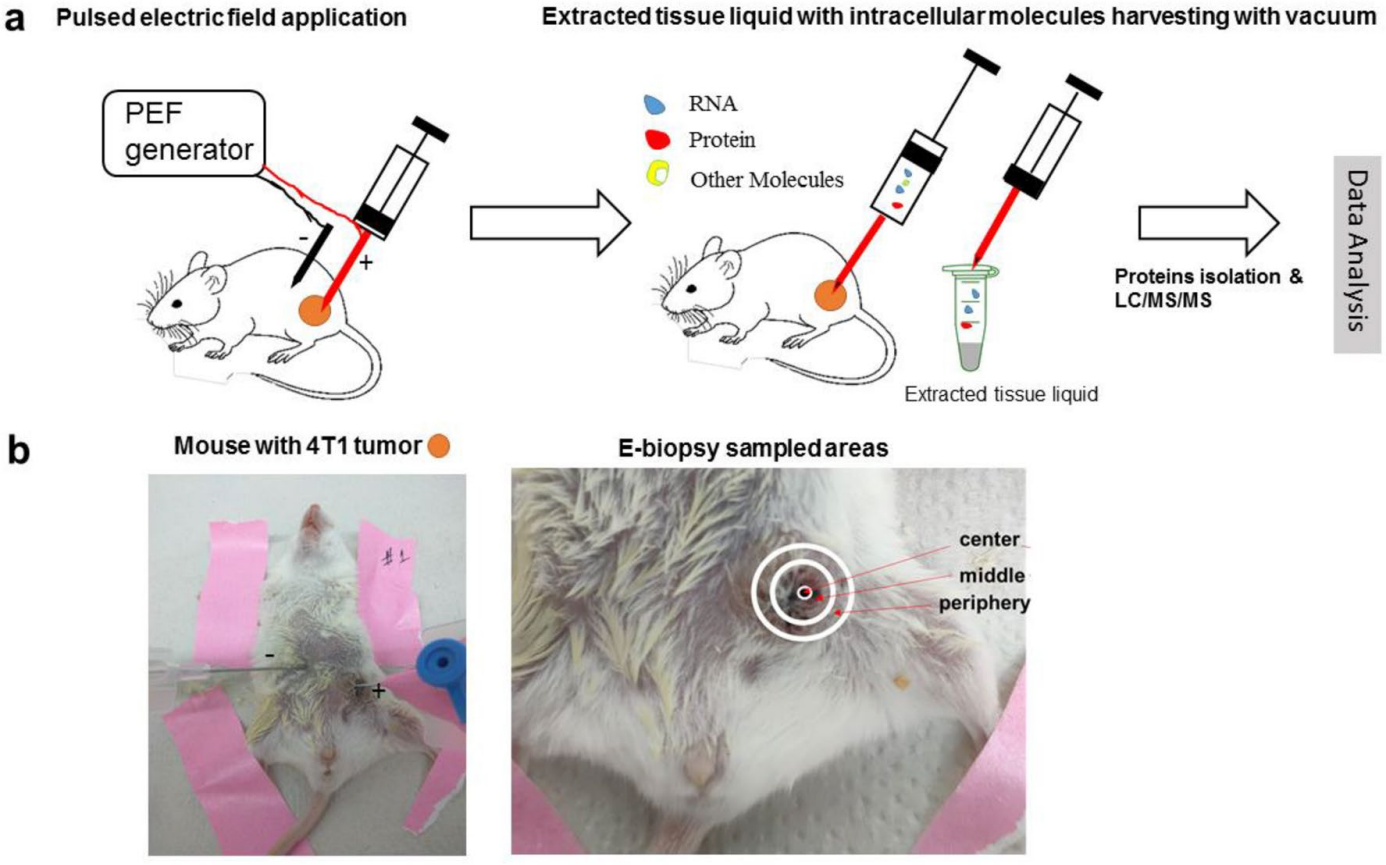
(**a**) Schematic depiction of molecular harvesting in vivo with e-biopsy. (**b**) Digital image of the e-biopsy procedure. The left image shows the insertion of the needle into areas of the tumor and the healthy breast. The right image shows the sampled locations: center, middle, and periphery. Two samples were taken from each location.

The e-biopsy method for molecular harvesting in vivo from solid tumors, using electroporation for cell membrane permeabilization, described for the first time in this work, is shown in Fig. 1a. First, the needles are inserted into the solid tissue. Second, the specific series of high-voltage pulsed electric fields (PEF) are applied to permeabilize the cell membrane with electroporation. Third, a vacuum is applied on the same needle through which the PEF pulses are delivered, to pump the released cellular content into the needle and the syringe. Next, the tissue extract is discharged from the syringe to the external buffer, and subjected to standard protocols for molecular analysis, including purification, separation, identification, and quantification. E-biopsy can be repeated in multiple positions in the same area or other areas of the tissue sample. In our study (Fig. 1b), 4T1 tumors (Fig. S1) were sampled 6 times each: 2 times at their centers, 2 times at their peripheries, and 2 times midway between the center and the periphery. Additional sampling was done in the healthy breast of the same animal. All animals survived the procedure, and no abnormal responses were observed. We found that the expression levels of proteins extracted from all locations in the tumor are highly correlated when comparing the location replicates (Table S1).

### E-biopsy proteomics distinguishes 4T1 tumor samples from healthy breast tissue

We first consider e-biopsy extracted proteomic profiles in the context of distinguishing between 4T1 tumor and healthy murine breast tissue, regardless of sampling site. Differential analysis of protein expression (paired two-sided t-test with *n* = 5, the two replicates from each location were averaged) was performed for three pairs of e-biopsy extracts: 4T1 tumor center versus healthy breast, 4T1 tumor periphery versus healthy breast, and 4T1 tumor middle versus healthy breast. We found 13 proteins harvested by e-biopsy (Table 1) strongly overexpressed (*p* value < 0.01, see “Methods” section) in all sampled locations in the tumor versus healthy breast (no underexpressed proteins were identified under the same criteria). An intersection of this size has a *p* value < 1.5e−06. These 13 proteins therefore represent an False Discovery Rate (FDR) of 3.5e−04. Moreover, releasing the *p* value cutoff to 0.05 results in a set of 242 (238 overexpressed and 4 under-expressed) differentially expressed proteins, corresponding to FDR of 2.3e−03. In further analysis we call these 242 proteins—potential 4T1 biomarkers.

**Table 1:** List of proteins extracted with e-biopsy that differentiate 4T1 tumor from healthy breast tissue samples for all tumor sampling locations.

#	Genes	Peripheral > Healthy breast			Middle > Healthy breast			Center > Healthy breast		
		*p* value	t-stats	Fold change	*p* value	t-stats	Fold change	*p* value	t-stats	Fold change
1	Vim	8.1E−03	4.9	2.2	1.7E−03	7.5	2.6	1.4E−03	7.9	2.3
2	Hnrnpa2b1	5.6E−04	10.0	5.0	5.8E−04	9.9	5.9	4.7E−04	10.5	5.0
3	Pabpc1	5.9E−03	5.3	4.7	8.6E−03	4.8	5.3	3.1E−03	6.4	4.8
4	Nucb1	7.4E−03	5.0	7.9	3.4E−03	6.2	5.5	2.5E−03	6.7	5.6
5	Serpinh1	3.7E−03	6.1	18.2	8.7E−03	4.8	13.0	7.6E−04	9.2	12.0
6	Hnrnpa1	2.9E−03	6.5	3.6	1.8E−03	7.3	3.4	9.5E−03	4.7	3.1
7	Slc25a24	3.2E−03	6.3	46.5	1.3E−03	8.1	69.5	2.4E−03	6.8	64.1
8	Plin3	9.9E−03	4.6	11.4	4.4E−03	5.8	10.8	9.7E−03	4.6	10.1
9	Hmgb1	3.8E−04	11.1	16.7	6.4E−03	5.2	18.4	3.7E−03	6.1	16.0
10	Rcn1	3.8E−03	6.0	152.1	1.3E−03	8.0	162.3	1.3E−03	8.0	125.3
11	Cnpy2	3.8E−03	6.0	7.8	9.3E−04	8.8	10.6	4.4E−03	5.8	9.8
12	Mat2b	1.5E−03	7.7	240.7	3.0E−04	11.7	254.4	2.5E−03	6.8	185.3
13	Bax	6.3E−03	5.2	84.9	1.2E−04	14.9	70.2	2.6E−03	6.7	63.0

This is a list of genes that are overexpressed in all 3 tumor locations together (Center/Midway/Peripheral; with both tumor location replicas averaged into a single value) compared to healthy breast samples (paired t-test *p* value < 0.01 at each location). A two-sided, paired t-test was applied to each of the 4,519 known proteins sampled from 5 mice via e-biopsy. No underexpressed proteins were identified using the same criterion, namely *p* < 0.01 in all three comparisons.

**Figure 2:**
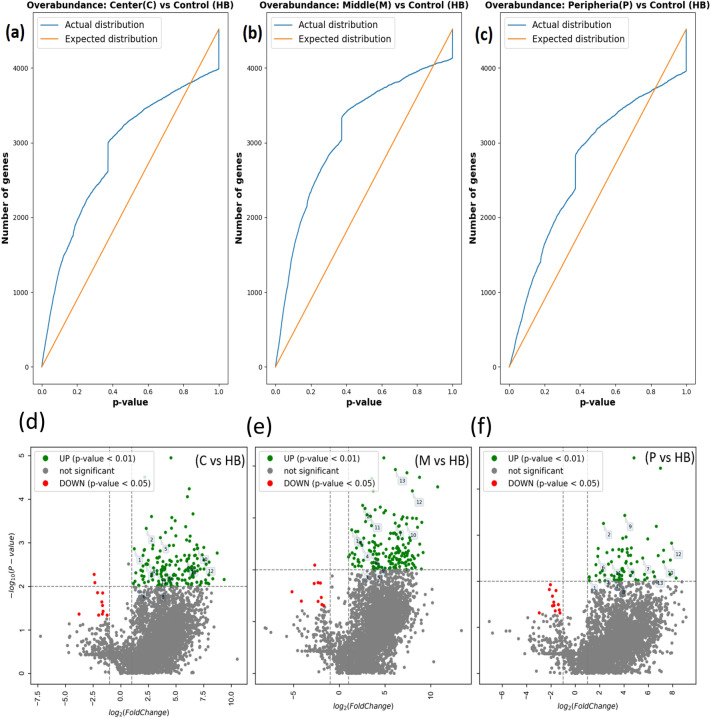
(**a**–**c**) Overabundance plots comparing the distribution of the protein differential expression (both over- and under-expression) *p* values between control (healthy breast) and 4T1 tumor samples. Total 5 mice and 4,519 proteins extracted by e-biopsy per sample were analyzed. (**a**) 4T1 center versus healthy breast. (**b**) 4T1 middle versus healthy breast. (**c**) 4T1 periphery versus healthy breast. (**d**,**e**) Volcano plots showing the fold change difference of protein expression. (**d**) 4T1 center versus healthy breast. (**e**) 4T1 middle versus healthy breast (**f**) 4T1 periphery versus healthy breast. Numbers on the plot address corresponding rows in Table 1.

The distribution of the observed differential expression scores, computed from comparing protein measurements in the healthy breast versus the different 4T1 tumor locations, is statistically significant, manifesting an overabundance of differentially expressed proteins. As shown in the overabundance and volcano plots (Fig. 2), there are more differentially expressed proteins observed in our data than expected under a random null model^51^.

Gene Ontology (GO) analysis of 4,519 e-biopsy-extracted proteins was used to further examine the differential expression between control (healthy breast) and three 4T1 tumor locations in terms of cellular processes, functions, and components. Here we present the most significant (*p* value < 1E−06 at all locations simultaneously) differentially regulated processes, functions, and components (Tables S2–S7) and discuss several interesting observations. All identified processes, functions, and components that are simultaneously enriched in all positions with *p* value < 1E−06 are presented online in https://github.com/GolbergLab/eBiopsy4T1.

Notably, the analysis of the GO terms revealed several significant (*p* value < 1E−06 in all locations) down-regulated cellular processes in 4T1 tumor (Table S2). Among them there are many immunoglobulin-related processes, which is consistent with earlier reports^52^, who found that intra-tumoral injection of allogeneic IgG combined with other factors induced nearly complete eradication of lung metastases from 4T1. The down-regulation of various peptidase inhibitors-related cellular functions (Table S3) in 4T1 is also consistent with previous works^53^. Moreover, the tumor’s extracellular component in all three locations was downregulated compared to the healthy breast (Table S4), which is expected in aggressive and invasive tumors such as 4T1^54^. Furthermore, we found that many biosynthesis-related cellular processes are up-regulated in 4T1 (Table S5), which is consistent with the tumor’s need for enhanced replication rates^55^. These findings are also supported by many upregulated cellular functions (Table S6) and cellular components (Table S7).

These data show that in vivo e-biopsy extraction of proteins yields statistically significant and biologically different profiles when comparing various locations in 4T1 tumors to healthy breast tissue in mice.

### In vivo e-biopsy supports mapping of 4T1 intratumor proteome spatial heterogeneity

To study the intratumor heterogeneity, we compared (two-sided, paired t-test) expression levels of proteins extracted in vivo by e-biopsy from three different tumor locations—center, midway, and periphery—in five animals. We found (Table S11) that 26 of 4,519 genes are significantly overexpressed, and 15 of 4,519 genes are significantly underexpressed in the center (compared to both other zones); 111 are overexpressed and 2 underexpressed in the middle; finally, 18 are overexpressed and 99 underexpressed at the periphery (significance here is defined by *p* value < 0.05). This represents FDR of 2.7e−01, 1.0e−01 and 9.7e−02 respectively (“Methods” section).

Next, we intersected the genes from the above analysis (41 from the center, 113 from the middle and 117 from the periphery) with the set of 242 potential 4T1 biomarkers (over/under-expressed in each of three 4T1 tumor locations compared to healthy breast with *p* value < 0.05, “Methods” section and text before Table 1). We found (Table 2) 2 such genes in the center, 3 in the middle and 3 in the periphery. This represents FDR of 7.1e−4, 4.7e−4 and 4.7e−4 respectively (“Methods” section).

**Table 2:** Potential 4T1 tumor biomarkers that significantly differ (“Methods” section) between three tumor areas.

Gene	Area	Direction	C versus M			C versus P			M versus P			C versus HB			M versus HB			P versus HB		
			*p* value	t-stats	fold	*p* value	t-stats	fold	*p* value	t-stats	fold	*p* value	t-stats	fold	*p* value	t-stats	fold	*p* value	t-stats	fold
Glrx	C	UP	2.5E−02	2.7	1.4	3.5E−02	2.5	1.5	Not relevant			2.2E−03	7.0	10.4	1.6E−02	4.0	7.7	4.9E−02	2.8	7.5
Rbbp7	M	UP	8.8E−03	− 3.3	7.3E−01	Not relevant			3.1E−02	2.5	1.4	3.5E−02	3.1	29.4	1.2E−02	4.3	43.5	4.4E−02	2.9	34.1
Ppme1	M	UP	1.4E−03	− 4.5	3.2E−02	Not relevant			7.5E−04	5.0	2.7	2.7E−02	3.4	20.9	1.3E−02	4.3	181.3	2.4E−02	3.6	116.9
Pkn1	M	UP	1.2E−02	− 3.1	1.1E−01	Not relevant			2.0E−02	2.8	23.4	4.9E−02	2.8	26.9	4.3E−03	5.8	188.0	3.6E−02	3.1	35.3
Ppme1	C	DOWN	1.4E−03	− 4.5	3.2E−02	4.7E−02	− 2.3	9.7E−02	Not relevant			2.7E−02	3.4	20.9	1.3E−02	4.3	181.3	2.4E−02	3.6	116.9
Prkcsh	P	DOWN	Not relevant			2.1E−02	2.8	1.4	2.1E−02	2.8	1.5	1.0E−02	4.6	23.6	2.6E−02	3.5	27.0	2.7E−02	3.4	18.9
Tra2a	P	DOWN	Not relevant			2.3E−02	2.7	3.1	1.8E−02	2.9	6.5	3.7E−02	3.1	69.2	1.8E−02	3.9	89.6	1.8E−02	3.9	41.3
Shoc2	P	DOWN	Not relevant			1.0E−02	3.2	9.0	3.4E−02	2.5	11.1	4.0E−03	5.9	147.2	2.0E−02	3.7	86.8	2.2E−02	3.7	41.0

C—Center; M—Middle; P—Periphery; HB—Healthy Breast.

**Figure 3:**
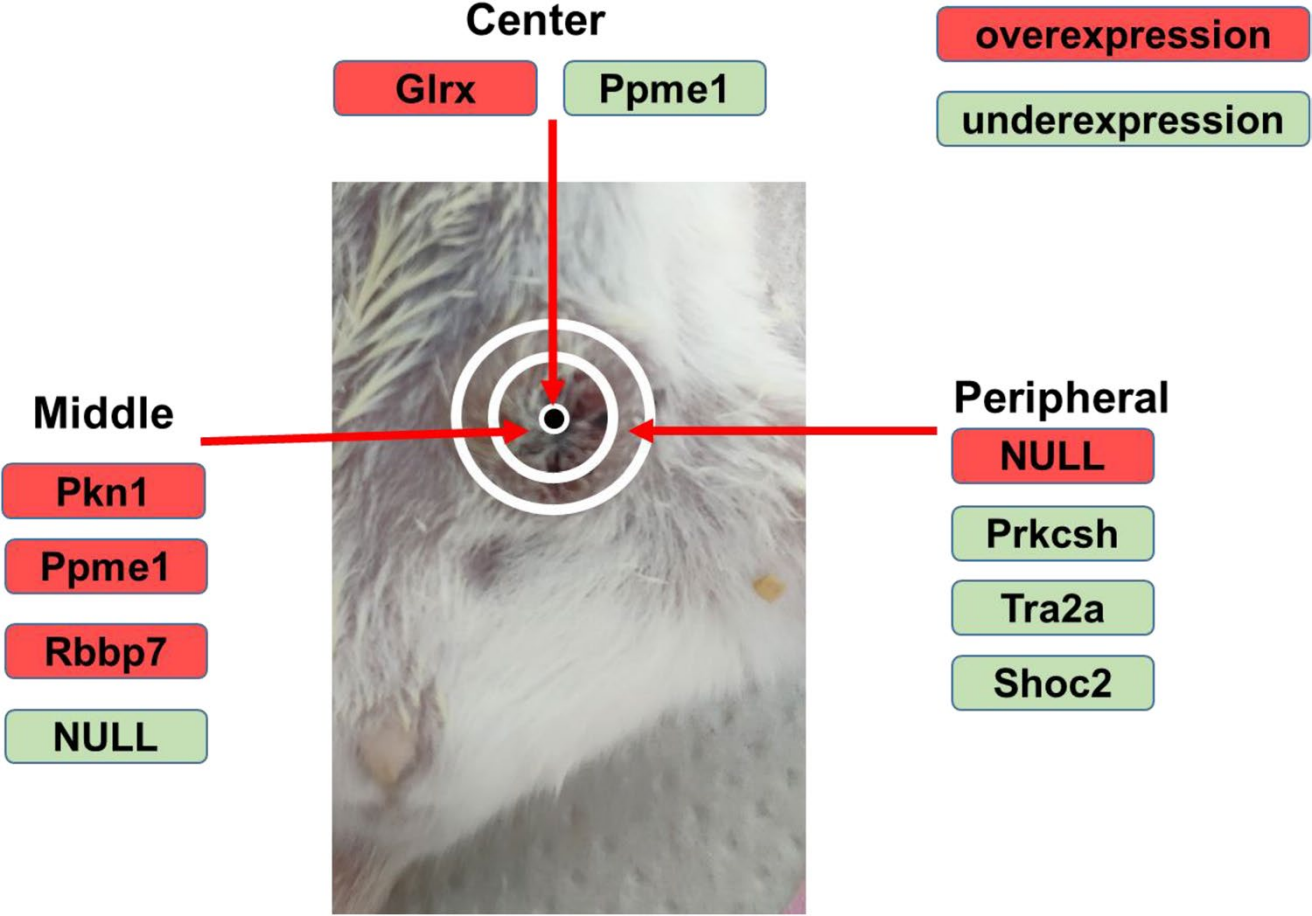
Molecular cartography of a 4T1 murine tumor, showing the potential tumor biomarker genes (*p* value < 0.05) that are differentially expressed in a single one of the three spatial zones simultaneously compared (*p* value < 0.05) to two others: center, middle, and periphery (Fig. 1). Overexpression direction (red) and underexpression direction (green) are indicated, NULL shows that no genes were funds under specific classification.

Specifically, we found (Table 2) that the gene Glrx is significantly overexpressed in the 4T1 tumor center compared to the tumor’s middle area and periphery (Fig. 3). We also found that Rbb7, Pkn1, and Ppme1 are overexpressed in the middle area of the tumor compared to its center and periphery (Fig. 3). No uniquely overexpressed potential tumor biomarker genes have been identified at the tumor periphery.

In the opposite direction, we found (Table 2) that the gene Ppme1 is significantly underexpressed in the 4T1 tumor center compared to tumor middle and tumor periphery (Fig. 3). Moreover, we found that Prkcsh, Tra2a, and Shoc2 are underexpressed at the tumor’s periphery compared to its center and mid-zones (Fig. 3). No uniquely underexpressed potential tumor biomarker genes have been identified in the middle area tumor zone.

**Figure 4:**
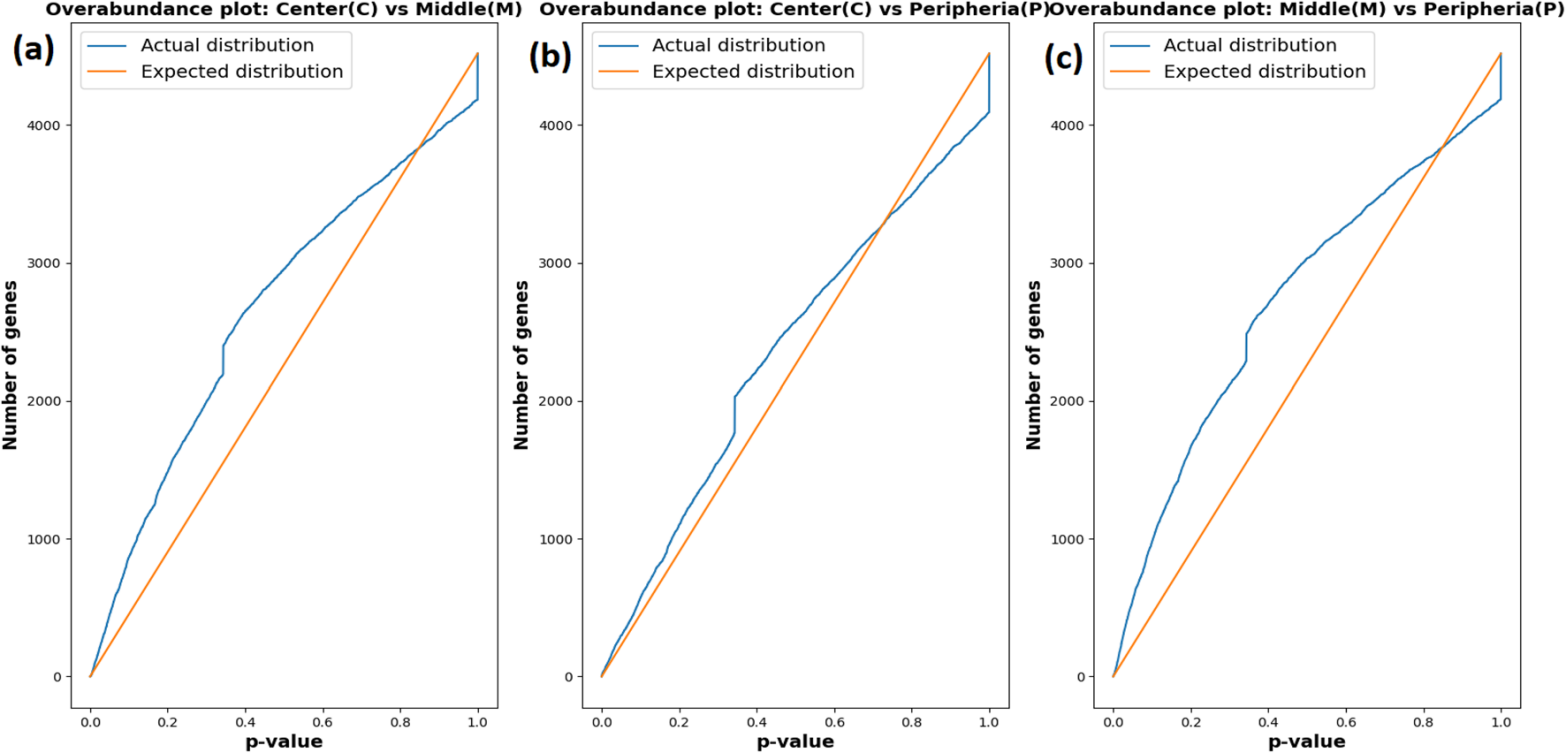
Overabundance plots comparing (“Methods” section) the distribution of the protein differential expression scores between 4T1 tumor locations. Total 5 mice and 4,519 proteins extracted by e-biopsy in 2 location replicas per sample were analyzed. (**a**) 4T1 center versus 4T1 periphery; (**b**) 4T1 center versus 4T1 middle; (**c**) 4T1 middle versus 4T1 periphery.

The distribution of the observed differential expression scores, computed from comparing protein measurements between 4T1 tumor locations, is statistically significant (Fig. 4). The overabundance plots show that more differentially expressed proteins are observed in our data than would be expected under a random null model ^51^. We also show that in vivo measurements obtained by e-biopsy are consistent with those obtained from the standard ex vivo lysis method (Supplementary information).

Gene Ontology (GO) analysis of 4,519 e-biopsy-extracted proteins was used to further examine the differential expression between all three locations in the 4T1 tumor in terms of various cellular processes, functions, and components. Here we present the most significant (*p* value < 1e−06 at each location simultaneously) differentially regulated processes, functions, and components (Table 3) and discuss several interesting observations. All the identified processes, functions, and components that differ with *p* value < 1e−06 are presented online in https://github.com/GolbergLab/eBiopsy4T1. In the comparison between Center and Periphery, no significant overexpressed function and components were identified in the Center. In the comparison between Middle and Periphery, no significant overexpressed processes, functions, and components were identified in the Middle (all three were overexpressed at the Periphery).

**Table 3:** GO terms significantly differentially regulated per each 4T1 tumor location.

Region	Direction	Description	Center *p* value	Middle *p* value	Peripheral *p* value
Center	High	PROCESS: blood coagulation		6.96E−09	4.94E−07
Center	High	PROCESS: coagulation		6.96E−09	4.94E−07
Center	Low	PROCESS: cytoplasmic translation		1.90E−11	5.60E−08
Center	Low	FUNCTION: structural constituent of ribosome		8.60E−11	5.36E−12
Center	Low	COMPONENT: cytosolic large ribosomal subunit		1.42E−11	1.54E−12
Center	Low	COMPONENT: ribosomal subunit		9.67E−11	1.02E−11
Center	Low	COMPONENT: ribosome		2.81E−11	4.06E−10
Center	Low	COMPONENT: cytosolic part		1.83E−11	2.52E−09
Center	Low	COMPONENT: cytoplasmic part		5.45E−09	2.33E−08
Center	Low	COMPONENT: ribonucleoprotein complex		2.78E−14	3.03E−08
Center	Low	COMPONENT: large ribosomal subunit		1.27E−07	1.59E−08
Center	Low	COMPONENT: intracellular		1.44E−09	5.09E−07
Periphery	High	COMPONENT: cytoplasmic part	2.33E−08	7.71E−07	

Two-sided, paired t-test *p* value < 1e−06 with two other tumor locations simultaneously.

Notably, in analyzing GO terms we found a significant decrease in ribosomal activity toward the 4T1 tumor’s central region. This finding is consistent with previous work that showed that tumorigenicity was associated with profound alterations in ribosomal biogenesis and function, leading to the decreased translation of mRNA of tumor suppressor p53 and the reduced control of translational fidelity^56^. Also, GO analysis yielded a significant increase in blood coagulation toward the center of the tumor, consistent with increased vacularization^57^.

The findings above show that proteomic profiling of in vivo e-biopsy samples can detect and potentially characterize 4T1 tumor heterogeneity. Specifically, the differences in protein expression profiles for the different sampled tumor regions are statistically significant. In addition, we identified enriched biological changes in cellular processes, functions, and components when comparing the 4T1 tumor’s center, middle, and periphery regions.

## Discussion

Current cancer treatment decisions are often based on the information obtained from an aspiration needle biopsy or a surgical excision. These excised samples are evaluated for histopathology. Sometimes molecular tests are used to obtain more precise diagnostic results^25^. Standard treatment of patients with metastatic disease is usually based on predictive biomarkers detected with the original biopsy, which often does not fully reflect the status of disease progression^25^. Moreover, multiple recent studies suggest that tumor biopsies may vastly underrepresent tumors’ heterogeneity and, therefore, may miss the drug-resistant clones^24,25,58,59^. In this work, we report a new method to probe tumors using molecular harvesting with electroporation, termed e-biopsy. Electroporation changes the permeabilization of the cell membrane, consequently increasing the accessibility of intracellular compounds^33^. In this work, we show that in vivo e-biopsy extraction of proteins yields a characteristic signature of 4T1 tumors versus healthy breast tissue in mice. We moreover show that point e-biopsy can detect various proteomic signatures in various geographical locations of the same tumor, thus increasing our understanding of the tumor sub-clonal spatial composition (Figs. 3, 4). As e-biopsy is potentially less aggressive than the current standard excision-based biopsy method, this technology can serve as a basis for new diagnostic approaches that will better address tumor heterogeneity, by probing tumors in multiple locations.

Molecular harvesting by e-biopsy is reproducible (Table S1) can distinguish between 4T1 tumor and healthy breast tissues, regardless of sampling location. We found 13 strongly overexpressed proteins (Table 1) in all sampled 4T1 tumor locations simultaneously. Some of these 13 proteins are known to have profound roles in breast cancer. For example, Vimentin (Vim), is considered a marker for epithelial-to-mesenchymal transition^60^. Long non-coding RNA (lncRNA), including VIM-AS1, and AGAP2-AS1 regulate Vim’s expression. Vim overexpression was reported in breast tumors in previous studies^61^. In addition, Hnrnpa21b overexpression was reported in endocrine-resistant LCC9 breast cancer cells^62^. Furthermore, triple-negative breast cancer patients face resistance to the drug trastuzumab by the active involvement of Polyadenylate-binding protein 1 (Pabpc1), expression of which is induced by overexpression of SNHG14^63^. For Serpinh1, also known as Hsp47, expression activation was reported during breast cancer development and progression^64^. Previous studies also demonstrated overexpression of hnRNPA1 during breast cancer progression^65^. Slc25a24, Cnpy2 and BAX overexpression was reported in breast cancer ^66^. Similar to our work, a recent study reported on the overexpression of Plin3 in the breast cancer tissues in comparison with healthy breast^67^. HMGB1 is considered a ubiquitous protein, which has a role as a nuclear cofactor in the regulation of transcription^68^. HMGB1 overexpression in breast cancer tissue indicates metastasis, TNM stage, and differentiation^69^. HMBG1 has a promising role in breast cancer management as it affects chemotherapy, immunotherapy, and radiotherapy^70^. Mat2b overexpression was observed in the triple negative breast cancer^71^. In summary, this comparison to literature analysis showed that 11 out of 13 proteins that were extracted with e-biopsy in vivo and measured as overexpressed have been reported as overexpressed in breast cancer in previous studies using other extraction and quantification methods.

Drug resistance is one of the major hurdles in cancer treatment^72,73^. There are several known resistance mechanisms, with heterogeneity in tumors being one of the most important amongst them^26^. Our work, using 4T1 as a model, shows that e-biopsy may help in charting and quantifying the heterogeneity in tumors, mapping over- and underexpressed genes spatially, and thereby leading to in vivo low resolution molecular tumor cartography. In Tables 2, 3, Fig. 3, and Table S11, we show an example of such a map based on differentially expressed genes in three spatial zones of a 4T1 tumor. In addition, the mass transport properties and time dynamics of the physical barriers to tumor drug delivery are very important in oncophysics as they are limiting the efficacy of some nanomedicines^74^. The proposed e-biopsy method could provide an additional dimension of information on the diffusion of molecules, which could assist in estimation of the time and space dynamics barriers to tumor drug delivery by providing a molecular cartography of sampled molecules with like nanodrugs transport properties.

In addition to individual gene expression analysis, we also performed a gene ontology (GO) enrichment analysis of the measured proteome extracted by e-biopsy and of the inferred differential proteomics. GO analysis revealed that overexpression and underexpression of biological processes (Table S2, S5), functions (Table S3, S6) and components (Table S4, S7) in 4T1 tumor, in comparison with the healthy breast samples, is similar to previously published GO studies on 4T1^75–78^, where the molecules were harvested with other methods. Analyzing the Gene Ontology for 4T1 in depth provides insight into differences between the tumor and the healthy breast, and the tumor’s various locations (Center, Middle, Periphery), and therefore suggests tumor heterogeneity for these studied samples (Tables 2, 3). Similar to our work, published literature on 4T1^75,77^ corroborates these findings. Altogether, the pathway enrichment analysis suggests that the proteomic profile detected by e-biopsy is corroborated by similar reports in the literature using other extraction methods. We therefore expect e-biopsy sampling to potentially yield biological information which is equivalent, at the level of gene sets or pathways, to that which would be inferred by other sampling technologies. Such information might be useful for other fields besides oncology, for example for regenerative medicine purposes for estimation of the state of the implant or its degree of biocompatibility^79,80^.

## Conclusions

In the current work we introduce e-biopsy, a novel tool for molecular harvesting in vivo using electroporation. E-biopsy has the potential to reduce the risks and morbidities of excision biopsy and to provide additional information and better profiling of the tumor and the probed environment in vivo. We demonstrate that e-biopsy enables the in vivo distinction between tumor and non-tumor samples and locations in the 4T1 mice model. Due to its minimal invasive nature, e-biopsy can potentially enable tumor sampling at multiple locations. We therefore hope that e-biopsy will potentially facilitate shedding light on the clonal subpopulation composition of tumors. This information on the tumor’s heterogeneity may be vitally important for higher precision personalized therapies. We therefore believe that e-biopsy represents a useful addition to the toolbox available to scientists and practitioners in their approach to treating cancer patients.

## Methods

All methods were performed in accordance with the relevant guidelines and regulations.

### Animals

All animal procedures were approved by the Israel National Council for Animal Experimentation (Study no. IL-19-3-114). Five 8-week-old female Balb/c mice weighing ~ 20 g were provided by the Science in Action, Ltd. CRO. The animals were housed in cages with access to food and water ad libitum and were maintained on a 12 h light/dark cycle at a room temperature of ~ 21 °C and a relative humidity of 30%-70%. All in vivo experiments were conducted by a professional veterinarian as per Israel National Council for Animal Experimentation guidelines and regulations. The study is reported in accordance with Animal Research: Reporting of In Vivo Experiments (ARRIVE 2.0 https://arriveguidelines.org/arrive-guidelines) guidelines.

### In vivo 4T1 tumor model

4T1 cell line was purchased from the American Type Culture Collection (Manassas, VA, USA). The cells were cultured in RPMI-1640 media with L-Glutamine supplemented with 10% fetal bovine serum (FBS), 0.11 mg/ml sodium pyruvate, 100 U/ml penicillin, and 100 μg/ml streptomycin (Biological Industries, Israel) at 37 °C in a humidified CO_2_ incubator. 4T1 cells were subcutaneously injected (0.5 × 10^6^ cells) into Balb/c female mice.

### Histology

Specimens were harvested immediately after the treatment and fixed in 10% formalin. Samples in plastic cassettes were dehydrated through ascending ethanol concentrations, transferred into xylene, and then paraffinized by an automated machine. Next, the samples were manually embedded into paraffin blocks. The paraffin blocks were sectioned at approximately 3–5 microns thickness. Sections were placed on glass slides. Slides were stained with Hematoxylin & Eosin (H&E) and covered by an automated machine.

### Immunohistochemistry

Paraffin blocks were sectioned at approximately 3–5 microns thickness. Sections were placed on SuperFrost Plus™ glass slides. Slides were incubated overnight at 64 °C. Slides were stained using the standard procedure in Ventana BenchMark Ultra automated slide stainer in combination with Ventana UltraView Universal DAB Detection Kit (Ventana, Roche Diagnostics cat #760-500). The slides were stained with the following antibodies: monoclonal mouse anti-Human Ki-67, clone MIB-1 (with Nuclear Dekloaker Dako as heat mediated antigen retrieval solution, cat# M7240), diluted 1:200, mouse anti-Human E-cadherin (with Nuclear Dekloaker Dako as heat mediated antigen retrieval solution Invitrogen, cat# #33-400, diluted 1:50, mouse anti-Human CK-7(with citrate as heat mediated antigen retrieval solution, Dako, cat#7019), diluted 1:200, mouse anti-Human CK-18 (with citrate as heat mediated antigen retrieval solution, Dako, cat#7010), diluted 1:3000, mouse anti-Human GATA-3 (with citrate as heat mediated antigen retrieval solution, Zytomed, cat#BMS054), ready to use.

### Pulsed electric field application for protein extraction in vivo

E-biopsy was performed with a 23G needle at 6 positions inside each tumor: 2 in the center, 2 at the periphery, and 2 in the middle between the center and the periphery (Fig. 1). An additional e-biopsy was performed on the normal breast of the same animal. The needle was connected to a cathode. The second 23G needle, connected to the anode, was held at a 1 cm distance from the first needle. The pulsed electric field was applied using the electric field pulse generator (BTX830, Harvard Apparatus, MA). Electroporation was performed using a combination of high-voltage short pulses with low-voltage long pulses as follows: 40 pulses 1000 V, 40 µs, 4 Hz, and 40 pulses 150Vcm^-1^, 15 ms, delivered at 4 Hz. After the PEF treatment, the liquids were extracted from the tissue to the needle applied to a vacuum with a 1.5 mL syringe. The liquids were immediately transferred to 1.5 ml tubes with 100 µl double distilled water (DDW).

### Isolating proteins from the pulsed electric field extracted juices

Proteins were isolated from the PEF extract using the protocol of the EZ- RNA II kit (Biological Industries Beit Haemek, Ltd, Israel). For protein isolation from PEF samples, the liquids from tissue after electroporation were added to 100 µl of DDW. Homogenizing solutions were not used in PEF samples; instead, phase separation solutions were directly added: 0.2 ml of water-saturated phenol and 0.045 ml of bathocuproine (BCP) buffer. Air-dried protein pellets were taken for proteomic analysis as described below.

### Isolating proteins from tissue using lysis buffer

Proteins were isolated using the protocol of the EZ- RNA II kit (Biological Industries Beit Haemek, Ltd, Israel). Tissue samples were homogenized in the Denaturing Solution (0.5 ml/50-100 mg tissue) using Bead Beater (Biospec, OK). Then 0.4 ml of water-saturated phenol and 0.09 ml of BCP were added. Air-dried protein pellets were taken for proteomic analysis as described below.

### Identifying and quantifying proteins using LC–MS/MS

#### Proteolysis

The samples were mixed in 8 M urea, 400 mM ammonium bicarbonate, 10 mM DTT, vortexed, sonicated for 5′ at 90% with 10–10 cycles, and centrifuged. The protein amount was estimated using Bradford readings. 20 µg protein from each sample was reduced at 60 °C for 30 min, modified with 37.5 mM iodoacetamide in 400 mM ammonium bicarbonate (in the dark at room temperature for 30 min), and digested in 2 M Urea and 100 mM ammonium bicarbonate with modified trypsin (Promega,WI) at a 1:50 enzyme-to-substrate ratio, overnight at 37 °C. An additional second digestion with trypsin was done for 4 h at 37 °C.

#### Mass spectrometry analysis

The tryptic peptides were desalted using C18 tips (Harvard Apparatus,MA), dried, and re-suspended in 0.1% formic acid. The peptides were resolved by reverse-phase chromatography on 0.075 X 180-mm fused silica capillaries (J&W) packed with Reprosil reversed-phase material (Dr. Maisch GmbH, Germany). The peptides were eluted with a linear 180-min gradient of 5 to 28%, 15 min gradient of 28 to 95%, and 25 min at 95% acetonitrile with 0.1% formic acid in water at flow rates of 0.15 μl/min. Mass spectrometry was performed using Q-Exactive Plus mass spectrometer (ThermoFischer Scientific, CA) in a positive mode using a repetitively full MS scan followed by collision-induced dissociation (HCD) of the 10 most dominant ions selected from the first MS scan.

The mass spectrometry data from all biological repeats were analyzed using MaxQuant software 1.5.2.8 versus the mouse proteome from the UniProt database with 1% FDR. The data were quantified by label-free analysis using the same software, based on extracted ion currents (XICs) of peptides, enabling quantitation from each LC/MS/MS run for each peptide identified in any of the experiments.

### Bioinformatics and statistical analysis

Data for 4,781 proteins was obtained from the mass spectrometry analysis, 4,519 of which were accompanied by valid protein and gene IDs. LFQ-intensity normalization for these 4,519 proteins, as obtained from MaxQuant 1.5.2.8, was used in all bioinformatics analyses.

### Inter-sample correlation analysis

Pearson and Spearman correlations were estimated between LFQ-intensity protein profiles of each sample with *scipy.stats.pearsonr* and *scipy.stats.spearmanr* functions respectively (Fig. S2, Table S9). Correlation coefficients were calculated separately per each mouse and then averaged.

To count the identified proteins (Table S8, Table S12) by each method (e-biopsy vs Lysis), we defined all proteins with strictly positive LFQ-intensity as existing within the specific sample (any sample in case of Location_MIX). If a protein was identified by e-biopsy/lysis only, it was marked as *uniquely captured by* e-biopsy/lysis. Otherwise (protein LFQ-intensity > 0 measured by both methods) it was marked as *simultaneously captured by both methods.* The value for Mouse_Average was derived as an average of all of the comparisons of samples within the same mouse.

### Differential expression analysis of control (healthy breast) and tumor (4T1) samples

The protein representations for control were constructed as 5D vectors based on e-biopsy LFQ-intensity measurements from healthy breast tissue in 5 mice. The protein representations for tumors were constructed as 5D vectors based on the average of two LFQ-intensity measurement replicas at specific 4T1 tumor locations from 5 mice. Paired two-tail Student *t*-test was performed with *scipy.stats.ttest_rel* function. Further, the overabundance comparison of the obtained distribution to the random model was performed^51^ (Fig. 2). 13 genes with Student *t*-test *p* values below 0.01 at each location simultaneously (73 such genes were identified in Peripheral, 160 in Middle and 164 in Center) were labelled as strongly overexpressed (no underexpressed genes for the same criteria were identified) in breast cancer tissue (Table 1). The intersection of this size has a *p* value below 1.15e−06, which is an upper limit based on Hypergeometric tail (HGT) with parameters (4519,160,73,13). These 13 proteins therefore represent an FDR of 4519∙(0.01^3)/13 = 3.5e−04. The same process at Student *t*-test *p* value of 0.05 results in a set of 242 genes (238 overexpressed and 4 under-expressed), corresponding to FDR of 4519∙(0.05^3)/242 = 2.3e−03. In the further analysis we call these 242 differentially expressed proteins—potential 4T1 biomarkers, specifically for the subsequent search for intra-tumor heterogeneity markers, particularly for filtering out Table 2 from Table S11.

Fold change in protein intensities was calculated as a geometric mean of ratios between measurements in tumor and in healthy breast in each mouse, while zero intensity was replaced with 50% of minimal observed LFQ-intensity in dataset to avoid numerical issues.

### Intratumor differential expression analysis

The protein representations for all tumor locations were constructed as 10D vectors based on e-biopsy LFQ-intensity measurements from two replicas at specific tumor locations in 5 mice. Paired two-tail Student *t*-test was performed with *scipy.stats.ttest_rel* function. Then, the overabundance comparison of the obtained distribution to the random model was performed^51^ (Fig. 4).

Genes with Student *t*-test *p* values below 0.05 with differential regulation in one zone compared to both others were extracted (Table S11). Specifically, for the up-regulation direction we tested for central location: both center > middle and center > periphery (26 such genes); for middle location: both center < middle and middle > periphery (111 such genes); and for peripheral location: both center < periphery and middle < periphery (18 such genes). The same analysis process was performed in the opposite directions for the down-regulation direction, resulting in 15, 2 and 99 genes respectively. These findings represent FDR of 4519∙(0.05^2)/(26 + 15) = 2.7e−01, 4519∙(0.05^2)/(111 + 2) = 1.0e−01 and 4519∙(0.05^2)/(18 + 99) = 9.7e−02 respectively. Next, the resultant genes were intersected with 242 potential biomarker candidates, and the results labelled as heterogeneously expressed in breast cancer tissue (Table 2, Fig. 3). We identified 2 such genes in the center, 3 in the middle and 3 in the periphery, that corresponds to FDR of 4519∙(0.05^2)∙(0.05^3)/2 = 7.1e−4, 4519∙(0.05^2)∙(0.05^3)/3 = 4.7e−4, 4519∙(0.05^2)∙(0.05^3)/3 = 4.7e−4 respectively.

Fold change in protein intensities was calculated as a geometric mean of ratios between measurements in two tumor areas in each mouse, while zero intensity was replaced with 50% of minimal observed LFQ-intensity in dataset to avoid numerical issues.

### Gene ontology analysis

The Gene Ontology analysis was performed on all proteins detected simultaneously in both tissues/locations of interest. These proteins were sorted as per their student *t*-test t-statistic values (in decreasing direction for overexpression and increasing direction for underexpression). Then cellular processes, functions, and components based on Gene Ontology (GO) were tested for significant (mHG *p* value < 1e−06) overabundance at the top of the obtained proteins list using Gorilla tool^81–83^.

GO analysis results for pairwise analysis of different 4T1 tumor Center, Periphery and Healthy breast appear online in https://github.com/GolbergLab/eBiopsy4T1.

## Supplementary Information

Supplementary Information 1.

Supplementary Information 2.

Supplementary Information 3.

Supplementary Information 4.

Supplementary Information 5.

## Data Availability

The authors hereby declare that all data supporting the findings of this study are available within the paper, its Supplementary Information and Online in https://github.com/GolbergLab/eBiopsy4T1.

## Abbreviations

e-biopsy: Molecular harvesting by electroporation
GO: Gene Ontology
mHG: Minimum-hypergeometric test
GOrilla: Gene Ontology enRIchment anaLysis and visuaLizAtion tool
FDR: False discovery rate
HGT: Hypergeometric tail
LFQ: Label-free quantitation
PEF: Pulsed electric field
H&E: Hematoxylin & Eosin
