# Supplementary material for: Nondestructive protein sampling with electroporation facilitates profiling of spatial differential protein expression in breast tumors in vivo
Source: Sci Rep. 2022 Sep 23;12:15835. doi: 10.1038/s41598-022-19984-x (PMC9508265; doi:10.1038/s41598-022-19984-x)
Supplement: Supplementary file 1 — Supplementary Information 1. [file 41598_2022_19984_MOESM1_ESM.docx]

**Supplementary information**

**
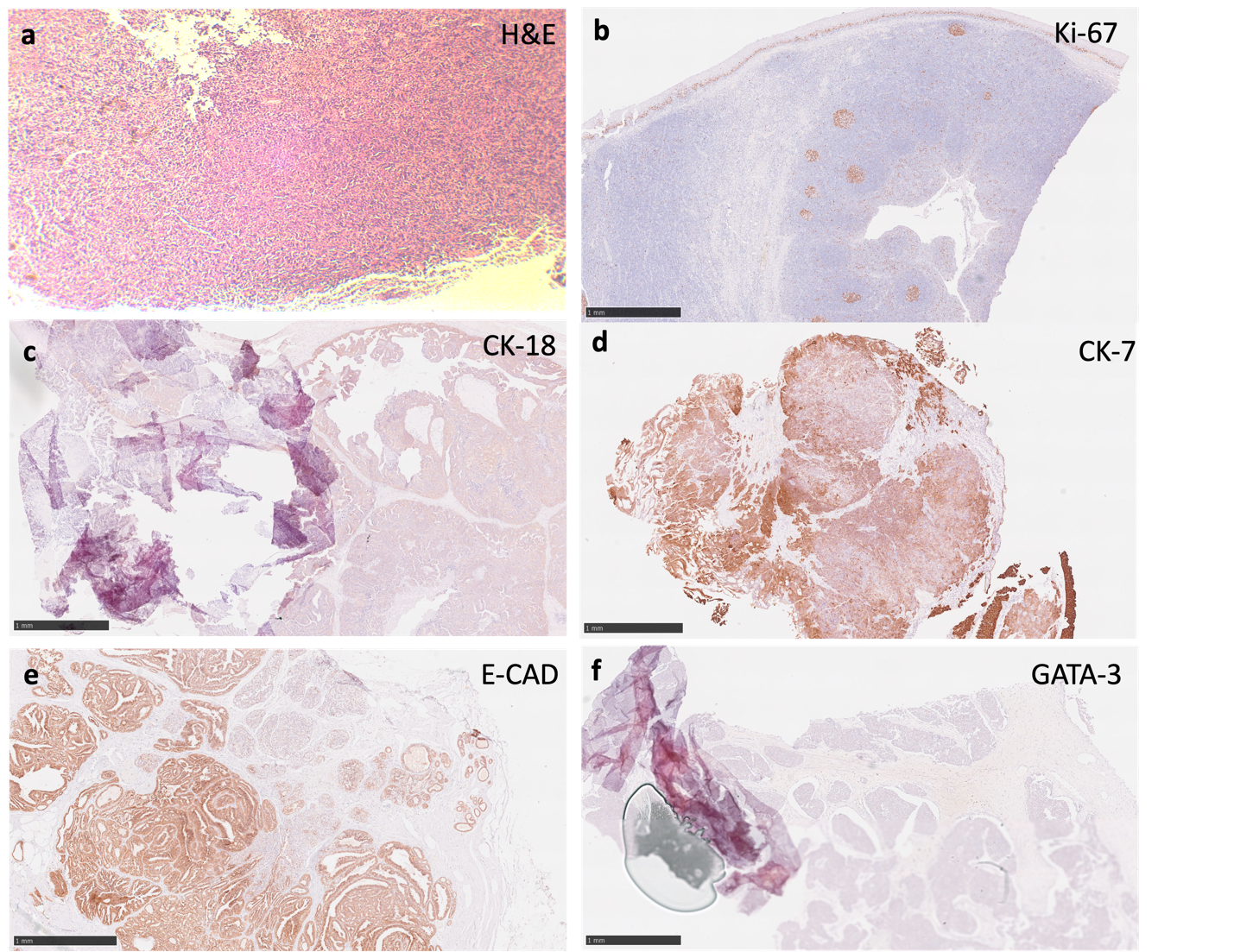
**

**Figure S1**. 4T1 breast tumor histological analysis. **a**. H&E staining. **b-f** immunohistochemical staining for **b.** Ki-67, **c.** CK-18, **d.** CK-7, **e.** E-CAD, **f.** GATA-3.

**Reproducibility of *in vivo* molecular harvesting with e-biopsy**

To study the reproducibility of our *in vivo* e-biopsy extraction method, we harvested liquids from 6 positions: 2 in the center, 2 in the middle, and 2 at the periphery (**Fig. 1**) from 4T1 tumors *in vivo* in 5 mice. In total, 4,262 proteins (with positive LFQ intensity in at least one e-biopsy sample) out of 4,519 total proteins were considered in this analysis. We found that the expression levels of proteins extracted from all locations in the tumor are highly correlated when comparing the location replicates.

**Table S1. E-biopsy reproducibility *in vivo.*** Average Pearson and Spearman correlation coefficients of the LFQ intensities of e-biopsy-extracted proteins, at various position replicates from 4T1 tumor, as quantified by LC-MS/MS. 4,262 proteins were considered from 5 mice (**Fig. 1)**. Correlation coefficients were calculated separately per each mouse and then averaged. All p-values < 10E-324.

| Location Replicates | Center | Middle | Periphery |
| --- | --- | --- | --- |
| Average Pearson Correlation | 0.928 | 0.944 | 0.845 |
| Average Spearman Correlation | 0.787 | 0.835 | 0.769 |

**Gene Ontology (GO) analysis of e-biopsy-extracted proteins for differential expression analysis between control (healthy breast) and three 4T1 tumor locations**.

Gene Ontology (GO) analysis of 4,519 e-biopsy-extracted proteins was used to further examine the differential expression between control (healthy breast) and three 4T1 tumor locations in terms of cellular processes, functions, and components.

**Table S2. GO Process terms significantly underexpressed in all 4T1 tumor locations vs healthy breast.** Two-sided, paired t-test p-value < 1E-06 for all 3 tumor locations simultaneously.

| Description | Center p-value | Middle p-value | Peripheral p-value |
| --- | --- | --- | --- |
| protein activation cascade | 3.22E-18 | 1.73E-20 | 5.40E-22 |
| complement activation | 8.94E-16 | 8.47E-19 | 3.35E-20 |
| humoral immune response | 4.67E-14 | 1.09E-14 | 1.41E-16 |
| complement activation, classical pathway | 7.83E-14 | 3.00E-15 | 3.27E-17 |
| immune response | 1.25E-12 | 6.54E-14 | 2.51E-13 |
| phagocytosis, recognition | 2.01E-11 | 3.20E-11 | 6.07E-13 |
| response to bacterium | 1.48E-13 | 5.56E-11 | 6.66E-13 |
| immunoglobulin production | 1.07E-11 | 9.08E-11 | 2.92E-12 |
| production of molecular mediator of immune response | 4.21E-11 | 3.17E-10 | 1.16E-11 |
| blood coagulation | 9.27E-10 | 3.91E-10 | 4.44E-10 |
| Coagulation | 9.27E-10 | 3.91E-10 | 4.44E-10 |
| defense response to bacterium | 1.25E-12 | 1.15E-09 | 1.55E-11 |
| Hemostasis | 5.76E-09 | 2.53E-09 | 1.37E-09 |
| immune system process | 1.02E-08 | 1.35E-08 | 1.92E-09 |
| defense response | 2.11E-08 | 1.25E-08 | 2.28E-10 |
| B cell receptor signaling pathway | 4.98E-08 | 3.14E-09 | 3.93E-09 |
| phagocytosis, engulfment | 1.60E-07 | 1.60E-07 | 1.50E-08 |
| activation of immune response | 1.71E-07 | 4.62E-07 | 5.06E-09 |

**Table S3. GO Function terms significantly underexpressed in all 4T1 tumor locations.** Two-sided, paired t-test p-value < 1E-06 for all 3 tumor locations simultaneously.

| Description | Center p-value | Middle p-value | Peripheral p-value |
| --- | --- | --- | --- |
| endopeptidase inhibitor activity | 1.25E-09 | 1.51E-08 | 2.24E-09 |
| peptidase inhibitor activity | 2.23E-09 | 2.21E-08 | 4.50E-09 |
| serine-type endopeptidase inhibitor activity | 3.93E-09 | 6.20E-08 | 2.27E-08 |
| endopeptidase regulator activity | 3.40E-08 | 1.24E-07 | 8.67E-08 |
| peptidase regulator activity | 1.82E-07 | 2.71E-07 | 5.09E-07 |

**Table S4. GO Component terms significantly underexpressed in all 4T1 tumor locations.** Two-sided, paired t-test p-value < 1E-06 for all 3 tumor locations simultaneously.

| Description | Center p-value | Middle p-value | Peripheral p-value |
| --- | --- | --- | --- |
| extracellular region part | 5.62E-21 | 9.83E-27 | 7.23E-28 |
| extracellular space | 3.25E-20 | 3.63E-25 | 1.09E-22 |
| extracellular region | 3.28E-15 | 1.66E-17 | 4.03E-18 |
| immunoglobulin complex | 4.51E-12 | 6.80E-13 | 8.62E-14 |
| immunoglobulin complex, circulating | 4.51E-12 | 6.80E-13 | 8.62E-14 |
| side of membrane | 2.04E-09 | 3.83E-07 | 3.22E-08 |
| cornified envelope | 9.30E-10 | 2.14E-08 | 6.68E-07 |

**Table S5. GO Process terms significantly overexpressed in all 4T1 tumor locations.** Two-sided, paired t-test p-value < 1E-06 for all 3 tumor locations simultaneously.

| Description | Center p-value | Middle p-value | Peripheral p-value |
| --- | --- | --- | --- |
| translation | 1.42E-14 | 9.60E-14 | 2.20E-19 |
| peptide biosynthetic process | 9.80E-14 | 8.59E-13 | 1.99E-17 |
| cellular macromolecule biosynthetic process | 4.96E-12 | 5.99E-15 | 9.25E-17 |
| amide biosynthetic process | 3.74E-11 | 2.47E-10 | 4.08E-14 |
| macromolecule biosynthetic process | 3.13E-10 | 3.06E-14 | 2.39E-15 |
| peptide metabolic process | 6.68E-12 | 1.22E-09 | 1.53E-13 |
| cellular nitrogen compound biosynthetic process | 1.90E-08 | 1.40E-09 | 1.15E-10 |

**Table S6. GO Function terms significantly overexpressed in all 4T1 tumor locations.** Two-sided, paired t-test p-value < 1E-06 for all 3 tumor locations simultaneously.

| Description | Center p-value | Middle p-value | Peripheral p-value |
| --- | --- | --- | --- |
| RNA binding | 7.48E-14 | 2.07E-21 | 4.56E-26 |
| structural constituent of ribosome | 1.65E-13 | 1.02E-17 | 1.39E-32 |
| nucleic acid binding | 1.79E-10 | 1.32E-21 | 2.92E-24 |
| heterocyclic compound binding | 4.95E-08 | 2.36E-14 | 1.46E-12 |
| organic cyclic compound binding | 2.47E-07 | 4.46E-13 | 2.79E-11 |
| mRNA binding | 9.43E-07 | 1.58E-09 | 4.56E-14 |

**Table S7. GO Component terms significantly overexpressed in all 4T1 tumor locations.** Two-sided, paired t-test p-value < 1E-06 for all 3 tumor locations simultaneously.

| Description | Center p-value | Middle p-value | Peripheral p-value |
| --- | --- | --- | --- |
| intracellular part | 1.46E-12 | 1.02E-13 | 1.26E-13 |
| ribosomal subunit | 6.01E-12 | 3.36E-16 | 2.33E-32 |
| cytosolic part | 3.61E-11 | 3.48E-15 | 4.12E-20 |
| ribosome | 2.42E-10 | 6.54E-15 | 1.21E-26 |
| ribonucleoprotein complex | 2.75E-10 | 6.04E-18 | 5.50E-27 |
| organelle | 3.28E-09 | 2.03E-09 | 1.42E-11 |
| intracellular organelle | 3.76E-09 | 2.45E-09 | 2.37E-12 |
| nucleus | 1.02E-08 | 1.15E-16 | 6.31E-15 |
| protein-containing complex | 1.36E-08 | 2.87E-14 | 6.26E-11 |
| cytoplasm | 1.39E-12 | 5.85E-08 | 1.31E-07 |
| intracellular non-membrane-bounded organelle | 3.47E-09 | 2.17E-07 | 1.42E-11 |
| non-membrane-bounded organelle | 4.92E-09 | 3.06E-07 | 1.58E-11 |

***In vivo* e-biopsy extraction of proteins yields profiles that strongly correlate with their matching lysis buffer extraction**

To investigate whether the *in vivo* protein extraction by e-biopsy faithfully represents the tumor proteomic profile (i.e., e-biopsy-based proteome is consistent with standard lysis-based proteome), we compared 6 proteomic profiles obtained by e-biopsy *in vivo* to 3 proteomic profiles obtained by standard lysis in excised tumors in 4 mice (sample from mouse #1 was lost during lysis extractions, therefore the analysis was performed for 4 animals here versus 5 animals in the other sections) (**Table S8**). In total, 4,511 proteins (with positive LFQ intensity in at least one e-biopsy or lysis sample) out of 4,519 total proteins were considered in this analysis. We found that the expression levels of proteins extracted from all locations strongly correlate between two methods (Pearson R of 0.831-0.934 (**Fig. S9**); Spearman R of 0.702-0.778; all p-values < 10E-324, **(Table S9**). This result suggests that *in vivo* e-biopsy of 4T1 tumors is a reliable method that reflects the proteomics of the sample as obtainable by lysis buffer from excised tissues.


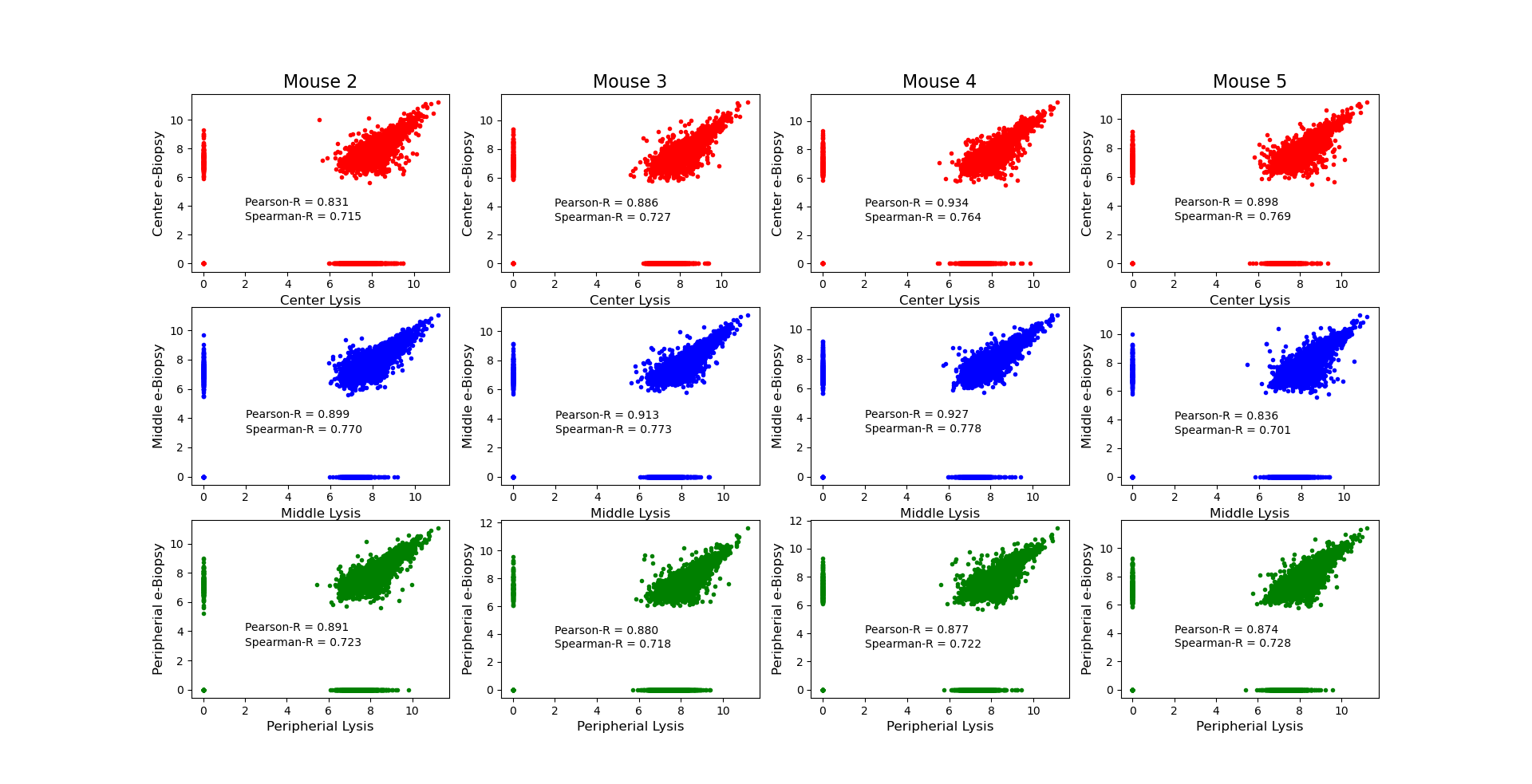


**c**

**b**

**a**

**
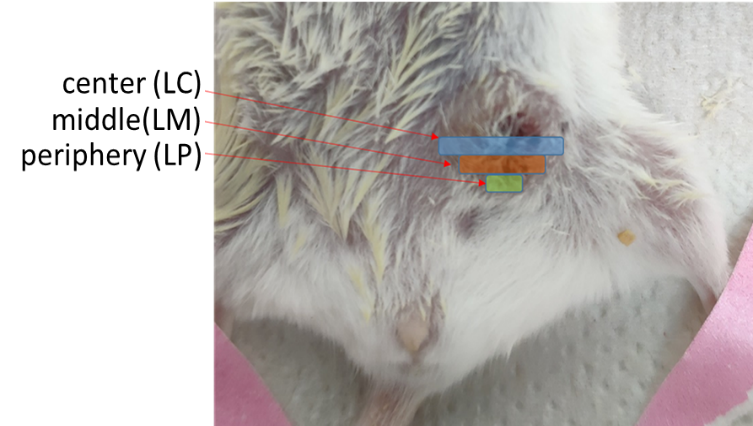
**
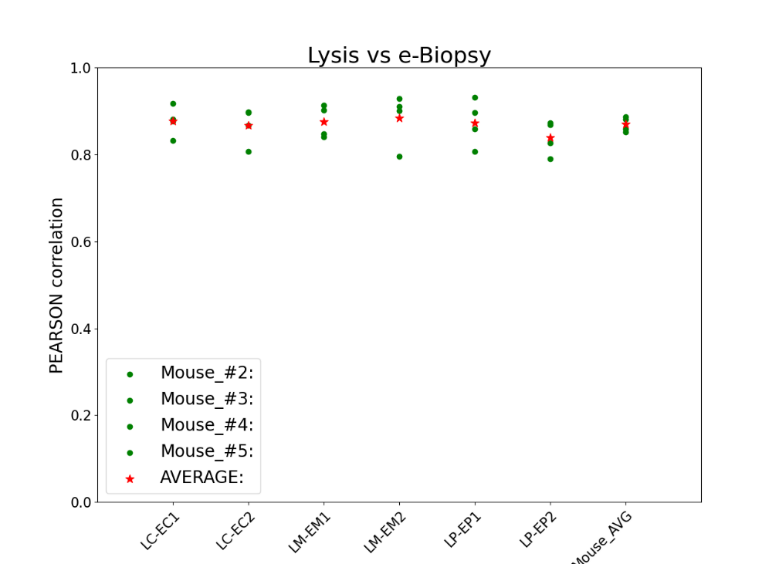


**Figure S2. a.** Scatter plot of *in vivo* expression (Log-10 of LFQ intensity of all relevant proteins at matched positions) measured with e-biopsy (average intensity between two reps) vs *ex vivo* Lysis buffer extraction in peripheral, middle, and center locations of 4T1 tumors in 4 animals. Pearson and Spearman R values are also shown. **b**. Comparison of protein expression intensity as extracted with e-biopsy *in vivo* vs tissue lysis buffer *ex vivo.* Pearson R correlation coefficient of the LFQ intensities of all relevant proteins at matched positions from 4T1 tumor as extracted with e-biopsy and tissue lysis buffer and quantified with LC-MS/MS. 4,511 (out of a total of 4,519) proteins were analyzed in 4 mice, from 6 e-biopsy locations in each mouse: 2 in the center (EC1 and EC2), 2 in the middle (EM1 and EM2), and 2 at the periphery (EP1 and EP2). Tissue lysis was performed from 3 extraction locations in each mouse: 1 in the center (LC), 1 in the middle (LM), and 1 at the periphery (LP)**.** All p-values < 10E-324. **c.** Image shows the areas from which the control samples were taken for protein extraction using a standard lysis buffer.

In **Table S8** and **Fig** **S3** we summarize the comparison between the proteins quantified (with LFQ-intensities) by one extraction method only vs both extraction methods. **Table S8** presents our findings on average for all samples (Mouse_Average) and on mixing all mouse samples together (considering all samples together, i.e., union of quantified protein sets Location_MIX), while the detailed per-sample data can be found in **Table S10**. Two interesting phenomena are observed here. First, there is a significant number of proteins identified by only one method, implying that lysis and e-biopsy technologies may represent complementary extraction methods^43^. Second, even though at each specific site, standard lysis technique extracted significantly more unique proteins than did e-biopsy (1,366 vs 171 uniquely identified proteins by lysis vs by e-biopsy respectively per each site on average (Mouse_Average)), this difference decreases significantly when considering all locations together (Location_MIX of proteins extracted at 3 lysis sites vs Location_MIX of proteins within 6 e-biopsy sites differ on average by only 572 vs 224 uniquely identified proteins by lysis vs by e-biopsy respectively). This second observation can be explained by the spatial molecular harvesting by e-biopsy together with the inherent heterogeneity of the tumor samples, which is less distinguishable by the standard lysis technique, which averages larger tissue volumes.

**Table S8** Summary of the number of unique proteins were captured by each method in each mouse on average and in all locations mixed. A full table describing each extraction site is at **Table S3**.

| **Methodology** | **Site** | **Mouse_2** | **Mouse_3** | **Mouse_4** | **Mouse_5** | **Average** |
| --- | --- | --- | --- | --- | --- | --- |
| **No. of proteins identified by Lysis only** | Mouse_Average: | 1,199 | 1,696 | 1,214 | 1,354 | 1,366 |
|  | Location_MIX: | 488 | 744 | 455 | 603 | 572 |
| **No. of proteins identified by e-biopsy only** | Mouse_Average: | 183 | 108 | 213 | 179 | 171 |
|  | Location_MIX: | 245 | 172 | 269 | 211 | 224 |
| **No. of proteins identified by both methods** | Mouse_Average: | 2,678 | 2,212 | 2,554 | 2,372 | 2,454 |
|  | Location_MIX: | 3,640 | 3,410 | 3,611 | 3,459 | 3,530 |

**
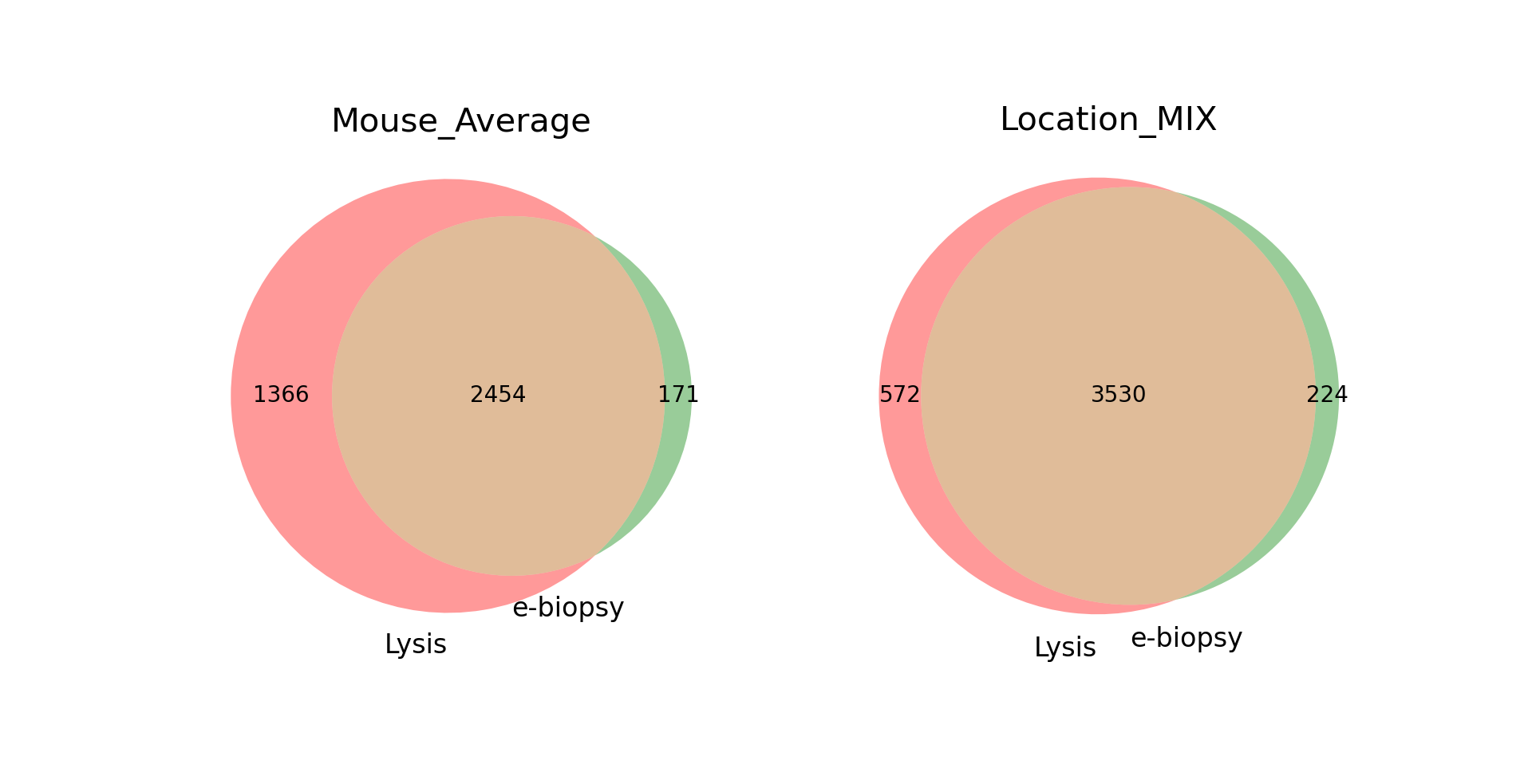
**

**Figure S3.** Per-mouse average number of unique proteins captured by each methods on average and in all locations mixed.

These findings are consistent with previous work on electroporation-based extraction of proteins from seaweed biomass. Our previous seaweed work showed that the electroporation-based extraction is selective and extracts proteins not discovered by other standard chemical methods^43,84^. The implication of these findings for tumor diagnostics may be addressed in further work, as e-biopsy could also serve to complement standard tissue lysis of excised samples.

The following tables are available online at <https://github.com/GolbergLab/eBiopsy4T1>

**Table S9. Spearman and Pearson correlations between proteins extracted with e-biopsy and lysis**

**Table S10. Proteins found only in lysis extract, only in e-biopsy extract, or in both extracts**

**Table S11. Proteins differently expressed in various locations in the tumor as extracted with e-biopsy**

**Table S12. Raw proteomic data**
